# Marine bacterial communities in the upper gulf of Thailand assessed by Illumina next-generation sequencing platform

**DOI:** 10.1186/s12866-020-1701-6

**Published:** 2020-01-23

**Authors:** Pongrawee Nimnoi, Neelawan Pongsilp

**Affiliations:** 1 Department of Microbiology, Faculty of Liberal Arts and Science, Kasetsart University, Nakhon Pathom, Thailand; 2 Department of Microbiology, Faculty of Science, Silpakorn University, Nakhon Pathom, Thailand

**Keywords:** The Gulf of Thailand, Bacterial community, Illumina next-generation sequencing, Land use, Marine microbiota

## Abstract

**Background:**

The total bacterial community plays an important role in aquatic ecosystems. In this study, bacterial communities and diversity along the shores of the Upper Gulf of Thailand were first characterized. The association between bacterial communities and types of land use was also evaluated.

**Results:**

The bacterial communities and diversity of seawater in the Upper Gulf of Thailand, with regard to types of land use, were first revealed by using Illumina next-generation sequencing. A total of 4953 OTUs were observed from all samples in which 554 OTUs were common. The bacterial communities in sampling sites were significantly different from each other. The run-off water from three types of land use significantly affected the community richness and diversity of marine bacteria. Aquaculture sites contained the highest levels of community richness and diversity, followed by mangrove forests and tourist sites. Seawater physicochemical parameters including salinity, turbidity, TSS, total N, and BOD_5_, were significantly different when grouped by land use. The bacterial communities were mainly determined by salinity, total N, and total P. The species richness estimators and OTUs were positively correlated with turbidity. The top ten most abundant phyla and genera as well as the distribution of bacterial classes were characterized. The *Proteobacteria* constituted the largest proportions in all sampling sites, ranging between 67.31 and 78.80%. The numbers of the *Marinobacterium*, *Neptuniibacter*, *Synechococcus*, *Candidatus* Thiobios, hgcI clade (*Actinobacteria*), and *Candidatus* Pelagibacter were significantly different when grouped by land use.

**Conclusions:**

Type of land use significantly affected bacterial communities and diversity along the Upper Gulf of Thailand. Turbidity was the most influential parameter affecting the variation in bacterial community composition. Salinity, total N, and P were the ones of the important factors that shaped the bacterial communities. In addition, the variations of bacterial communities from site-to-site were greater than within-site. The *Proteobacteria*, *Bacteroidetes*, *Actinobacteria*, *Cyanobacteria*, *Verrucomicrobia*, *Euryarchaeota*, *Planctomycetes*, *Firmicutes*, Deep Sea DHVEG-6, and *Marinimicrobia* were the most and common phyla distributed across the Upper Gulf of Thailand.

## Background

The Gulf of Thailand locates from 6°N to 13°30′N latitude and 99°E to 104°E longitude. It is a semi-enclosed tropical marine embayment which is surrounded by the land masses of Thailand, Cambodia, Vietnam, and Malaysia [1]. It constitutes a part of the South China Sea and is characterized as an estuary of drowned river valley. It is divided into the Upper and Lower Gulf based on its geography. The Upper Gulf has an inverted U-shape with the area of approximately 10,000 square km and the deepest point of approximately 40 m. The Upper Gulf is the catchment basin of four large rivers including the Chaopraya River, the Thachin River, the Bangpakong River, and the Maeklong River [2]. The coastal areas of the Upper Gulf are composed of agricultural areas, aquaculture areas, mangrove forests, swamp forests, forests, industrial areas, and urban areas [3], therefore it is of importance for natural resources, environment, and public health. Coastal seawater of the Upper Gulf of Thailand is utilized for environmental preservation, coral conservation, conservation of natural resources, aquaculture, fishery, water sport, recreation, transportation, and industry. Despite its importance, the total microbiota has not been investigated. The total bacterial community, which plays an important role in aquatic ecosystems, should be considered as a rigorous criterion for water quality to promote sustainable development. It is important to evaluate changes in the microbial community structure in aquatic systems because the microbial community is the foundation of biogeochemical cycles and pollutant biodegradation [4]. In particular, there is growing interest in the role of marine microorganisms that inhabit extreme habitats in biogeochemical processes, pollution, and health. Thermophiles, halophiles, alkalophiles, psychrophiles, piezophiles, and polyextremophiles have been isolated from marine environments. Marine environments represent the richest source of new genes, enzymes, and natural products [5]. Marine bacterial community structure is affected by several factors such as inorganic nutrient concentration [6], N [7, 8], P [7], change in season, adjacent habitat [9], depth [8, 10], oxygen [10], protist predation pressure [11], salinity [7, 8, 12], dominance of algae, particulate organic carbon, Si (OH)_4_ [8], human disturbance, and sand mining activity [13].

To explore the total bacterial community in the environment, high-throughput next-generation sequencing (NGS) technology of the taxonomically informative 16S rRNA gene provides the most powerful approach because it enables the classification of individual reads to specific taxa [14]. The contemporary advances in NGS have not only enabled finer characterization of bacterial genomes but also provided deeper taxonomic identification of complex microbiomes [15]. NGS approach was employed to survey microbial communities from several marine environments such as the Gulf of Mexico [16, 17], the Georgetown Coast, Malaysia [18], Malipo Beach, South Korea [19], the Canadian Arctic archipelago [8], and the South Sea, Korea [13].

In this study, we investigated 1) the marine bacterial communities at nine sites along the shores of the Upper Gulf of Thailand by using Illumina NGS of the V4 variable region of 16S rRNA gene; 2) the association between bacterial community structures and three types of land use including mangrove forests, tourist sites, and aquaculture sites; 3) effect of seawater physicochemical parameters on the abundance of specific taxa; and 4) the correlation between seawater physicochemical parameters and types of land use.

## Results

### Seawater physicochemical parameters

**Fig. 1:**
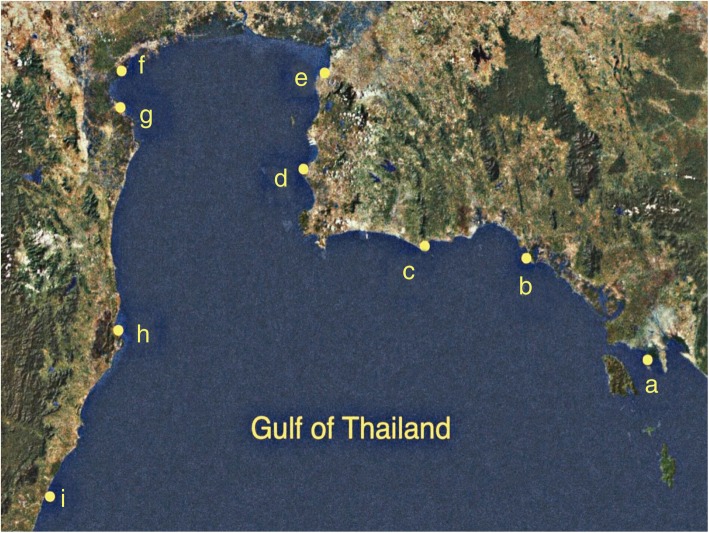
Map of sampling sites along the shores of the Upper Gulf of Thailand Retrieved from a development of drought risk analysis platform of Kasetsart University Research and Development Institute (KURDI) (http://csrs.ku.ac.th). Site **a**, mangrove forest at Black Sand Beach; **b**, mangrove forest at Kungkrabaen Bay; **c**, tourist site at Suanson Beach; **d**, tourist site at Pattaya Beach; **e**, aquaculture site at Angsila old market; **f**, aquaculture site at Donhoylhod; **g**, aquaculture site at Bangtaboon Bay; **h**, mangrove forest at Pranburi forest park; **i**, tourist site at Wanakorn Beach

Nine sampling sites in seven provinces along the shores of the Upper Gulf of Thailand, over a distance of approximately 769.97 km, are shown in Table 1 and Fig. 1. Seawater parameters including temperature, pH, salinity, turbidity, total suspended solid (TSS), total N, total P, and five-day biochemical oxygen demand (BOD_5_) at nine sampling sites are shown in Table 2. Temperatures and pH values of seawater samples among nine sampling sites ranged from 27 °C to 31 °C and 6.7 to 7.5, respectively. Salinity (presented as % NaCl) of all sites were 4.0, except sites F (aquaculture site at Donhoylhod) and G (aquaculture site at Bangtaboon Bay) that were 3.0 and 1.0, respectively. Turbidity, TSS, total N, and total P of all sites ranged from 2.32 ± 0.03 to 102.00 ± 1.00 nephelometric turbidity units (NTUs), 22.00 ± 0.80 to 177.66 ± 5.50 mg/l, 0.13 ± 0.00 to 1.30 ± 0.13 mg/l, and 0.03 ± 0.00 to 0.10 ± 0.01 mg/l, respectively. Site G (aquaculture site at Bangtaboon Bay) had the highest turbidity, TSS, total N, and total P which were significantly different from those of other sites. On the contrary, site A (mangrove forest at Black Sand Beach) was the only site that had significantly lowest turbidity. Sites A (mangrove forest at Black Sand Beach), B (mangrove forest at Kungkrabaen Bay), C (tourist site at Suanson Beach), and I (tourist site at Wanakorn Beach) shared the lowest rank of TSS which was significantly different from that of other sites. Site C (tourist site at Suanson Beach) contained the lowest amount of total N which was not significantly from that of site D (tourist site at Pattaya Beach). Sites A (mangrove forest at Black Sand Beach) and E (aquaculture site at Angsila old market) contained the lowest amounts of total P which were not significantly different from those of sites B and C. BOD_5_ values of all sites ranged between 0.90 ± 0.00 and 3.76 ± 0.23 mg/l. Site B (mangrove forest at Kungkrabaen Bay) had the highest BOD_5_ value, differing significantly from that of other sites. On the contrary, sites F (aquaculture site at Donhoylhod) and I (tourist site at Wanakorn Beach) had the lowest BOD_5_ values.

**Table 1:** Site locations, sampling dates and land use

Site	Sampling date	Place	District, Province	Latitude	Longitude	Distance (km)	Land use
A	11/10/2018	Black Sand Beach	Laemngop, Trat	12.169 N	102.406E	0.00	Mangrove forest
B	11/10/2018	Kungkrabaen Bay	Thamai, Chanthaburi	12.573 N	101.902 E	123.57	Mangrove forest
C	11/10/2018	Suanson Beach	Mueang, Rayong	12.458 N	101.473 E	190.51	Tourist site
D	11/10/2018	Pattaya Beach	Banglamoong, Chonburi	12.936 N	100.883 E	326.22	Tourist site
E	11/10/2018	Angsila old market	Mueang, Chonburi	13.341 N	100.926 E	388.60	Aquaculture site
F	12/01/2018	Donhoylhod	Meung, Samutsongkhram	13.361 N	100.022 E	527.64	Aquaculture site
G	12/01/2018	Bangtaboon Bay	Banlaem, Phetchaburi	13.264 N	99.945 E	548.77	Aquaculture site
H	12/01/2018	Pranburi forest park	Pranburi, Prachuapkhirikhan	12.412 N	99.981 E	659.23	Mangrove forest
I	12/01/2018	Wanakorn Beach	Thubsakae, Prachuapkhirikhan	11.635 N	99.703 E	769.97	Tourist site

**Table 2:** Seawater parameters and alpha diversity indices of bacterial communities and richness among all samples collected and consolidated by site

Site	Air temp (°C)	Seawater parameter data								Bacterial diversity indices^a^				
		Seawater temp. (°C)	pH	% NaCl	Turbidity (NTU)^a^	TSS (mg/l)^a^	Total N (mg/l)^a^	Total P (mg/l)^a^	BOD_5_ (mg/l)^a^	OTUs	Chao1	ACE	Shannon	Simpson
A	28	29	7.0	4.0	10.96± 0.25d^b^	22.00± 0.80a	0.42 ± 0.06bc	0.03± 0.01a	1.90 ± 0.00de	5854	1940.77 ± 69.19c	2022.59 ± 68.24c	7.33 ± 0.06bc	0.97 ± 0.00b
B	25	28	7.2	4.0	12.60 ± 0.26e	26.66± 1.00a	0.42 ± 0.06bc	0.04± 0.01ab	3.76 ± 0.23f	4968	1631.89 ± 41.68ab	1675.02 ± 47.72ab	7.23 ± 0.04b	0.97 ± 0.00b
C	30	29	7.1	4.0	10.80 ± 0.17d	27.53± 0.30a	0.13± 0.00a	0.04± 0.00ab	1.40± 0.10bc	4829	1588.03± 25.36ab	1649.36± 37.50ab	7.46± 0.06 cd	0.97± 0.00b
D	26	31	7.4	4.0	24.80 ± 0.30f	65.60± 1.74c	0.21± 0.06ab	0.06± 0.00bc	1.43± 0.05bc	5023	1630.61± 7.82ab	1708.45± 24.40ab	6.91± 0.05a	0.96± 0.00a
E	27	30	7.4	4.0	3.76 ± 0.20b	43.86 ± 1.00b	0.54 ± 0.07 cd	0.03 ± 0.00a	1.66 ± 0.05 cd	5284	1813.01 ± 209.44bc	1851.33 ± 195.23bc	7.40 ± 0.01c	0.97 ± 0.00b
F	31	27	6.7	3.0	91.33 ± 0.57 g	165.33 ± 5.03d	0.67 ± 0.09d	0.07 ± 0.00c	1.16 ± 0.11ab	6016	1946.48 ± 177.06c	2037.32 ± 218.82c	7.60 ± 0.07d	0.98 ± 0.00c
G	26	30	7.5	1.0	102.00 ± 1.00 h	177.66 ± 5.50e	1.30 ± 0.13e	0.10 ± 0.01d	2.03 ± 0.05e	5000	1622.41 ± 66.51ab	1680.02 ± 88.45ab	7.84 ± 0.00e	0.98 ± 0.00c
H	29	28	6.9	4.0	7.86 ± 0.15c	36.66 ± 1.52b	0.42 ± 0.06bc	0.06 ± 0.00c	1.26 ± 0.11bc	4696	1545.04 ± 16.43ab	1608.19 ± 31.63ab	7.00 ± 0.06a	0.97 ± 0.00b
I	31	30	7.0	4.0	2.32 ± 0.03a	28.33 ± 0.57a	0.38 ± 0.00bc	0.07 ± 0.01c	0.90 ± 0.00a	4246	1383.67 ± 77.21a	1443.29 ± 63.56a	6.92 ± 0.06a	0.97 ± 0.00b

All sites were sampled in triplicate (*n* = 3)

^a^Values are the means from three samplings ± standard deviations

^b^Values with the same letters within a column are not significantly different according to Tukey’s test

In this study, the results in Table 3 show that air temperature, pH, and total P were not significantly different when grouped by land use (*P* = 0.27, 0.35, and 0.13). On the contrary, seawater temperature, % NaCl, turbidity, TSS, total N, and BOD_5_ were significantly different when grouped by land use (*P* = 0.00, 0.01, 0.00, 0.00, 0.00, and 0.01). Turbidity, TSS, and total N of aquaculture sites were highest, differing significantly from those of other types of land use. BOD_5_ value of mangrove forests was highest, differing significantly from that of tourist sites. Aquaculture sites had the lowest values of % NaCl that were significantly different from those of other types of land use.

**Table 3:** Physicochemical parameters analyzed based on type of land use

Type of land use	Air temp. (°C)*	Seawater parameter data*							
		Seawater temp. (°C)	pH	%NaCl	Turbidity (NTU)	TSS (mg/l)	Total N (mg/l)	Total P (mg/l)	BOD_5_ (mg/l)
Mangrove forests	27.33 ± 1.80a^**^	28.33 ± 0.50a	7.03 ± 0.13a	4.00 ± 0.00b	10.47 ± 2.09a	28.44 ± 6.56a	0.42 ± 0.06a	0.04 ± 0.01a	2.31 ± 1.13b
Tourist sites	29.00 ± 2.29a	30.00 ± 0.86b	7.16 ± 0.18a	4.00 ± 0.00b	12.64 ± 9.83a	40.48 ± 18.85a	0.24 ± 0.11a	0.05 ± 0.01a	1.24 ± 0.26a
Aquaculture sites	28.00 ± 2.29a	29.00 ± 1.50ab	7.20 ± 0.37a	2.66 ± 1.32a	65.70 ± 46.68b	128.95 ± 64.50b	0.84 ± 0.36b	0.07 ± 0.03a	1.62 ± 0.38ab
*p*-value*** (between groups)	0.27	0.00	0.35	0.01	0.00	0.00	0.00	0.13	0.01

*Values are the means of three samplings from each location ± standard deviations

^**^Values with the same letters within a column are not significantly different according to Tukey’s test

***p-values < 0.05 are considered significant

### Sequence analyses and diversity indices

A total of 2,478,774 raw reads were obtained from 27 DNA samples (3 replicates/sampling site). After tag merge and quality control, a total of 2,425,463 clean tags (97.85% of raw reads) were obtained. After that, potential chimera tags were removed with the UCHIME algorithm, resulting in a total of 2,177,667 taxon tags. The tags with ≥97% similarity were grouped into the same operational taxonomic units (OTUs). A total of 4953 OTUs were observed from all samples, with a mean Good’s coverage of 99.00 ± 0.00%. ACE (abundance-based coverage estimator) and Chao1 that represent richness as well as Shannon-Weaver and Simpson that indicate diversity were analyzed (Table 2). When measured by ACE and Chao1, samples collected were significantly different when grouped by land use (*P* = 0.02 and 0.01) (Table 4). Aquaculture sites contained the highest community richness, followed by mangrove forests and tourist sites, respectively. The indices of community diversity assessed by Shannon and Simpson also exhibited that samples collected were significantly different when grouped by land use (*P* = 0.00 and 0.00). The bacterial community diversity of aquaculture sites was significantly highest, followed by mangrove forests and tourist sites, respectively (Table 4).

**Table 4:** Community richness and community diversity indices analyzed based on type of land use

Type of land use	Community richness		Community diversity	
	Chao1	ACE	Shannon	Simpson
Mangrove forests	1705.90 ± 184.77ab**	1768.60 ± 197.76ab	7.19 ± 0.15a	0.97 ± 0.00a
Tourist site	1534.10 ± 121.39a	1600.37 ± 126.66a	7.10 ± 0.27a	0.97 ± 0.00a
Aquaculture site	1739.97 ± 199.51b	1856.22 ± 217.73b	7.61 ± 0.19b	0.98 ± 0.00b
*p*-value*** (between groups)	0.01	0.02	0.00	0.01

*Values are the means of three samplings from each location± standard deviations

**Values with the same letters within a column are not significantly different according to Tukey’s test

***p-values < 0.05 are considered significant

The bacterial richness (ACE and Chao1) of site F (aquaculture site at Donhoylhod) was highest, followed by sites A (mangrove forest at Black Sand Beach) and E (aquaculture site at Angsila old market), respectively. The bacterial richness of site I (tourist site at Wanakorn Beach) was lowest. Higher Shannon-Weaver and Simpson indices indicate greater bacterial diversity. The highest values of both indices were found in site G (aquaculture site at Bangtaboon Bay), followed by sites F (aquaculture site at Donhoylhod) and C (tourist site at Suanson Beach), respectively. The lowest values were found in site D (tourist site at Pattaya Beach).

### Illumina NGS and bacterial community structure

Rarefaction analysis was used to standardize and compare taxon richness among samples and to identify whether the samples were randomly selected. According to the rarefaction curves of samples (Additional file 1: Figure S1), all of the samples were randomly collected. Moreover, aquaculture sites exhibited the steepest rarefaction curves, indicating the highest taxon richness, while tourist sites exhibited the most gradual curves. As shown in a Flower display (Additional file 2: Figure S2), 554 OTUs were common in all sampling sites. Site G (aquaculture site at Bangtaboon Bay) had the highest unique OTUs (259 OTUs), followed by sites A (mangrove forest at Black Sand Beach) and F (aquaculture site at Donhoylhod), respectively, whereas sites H (mangrove forest at Pranburi forest park) and I (tourist site at Wanakorn Beach) had the equally lowest unique OTUs (58 OTUs).

**Fig. 2:**
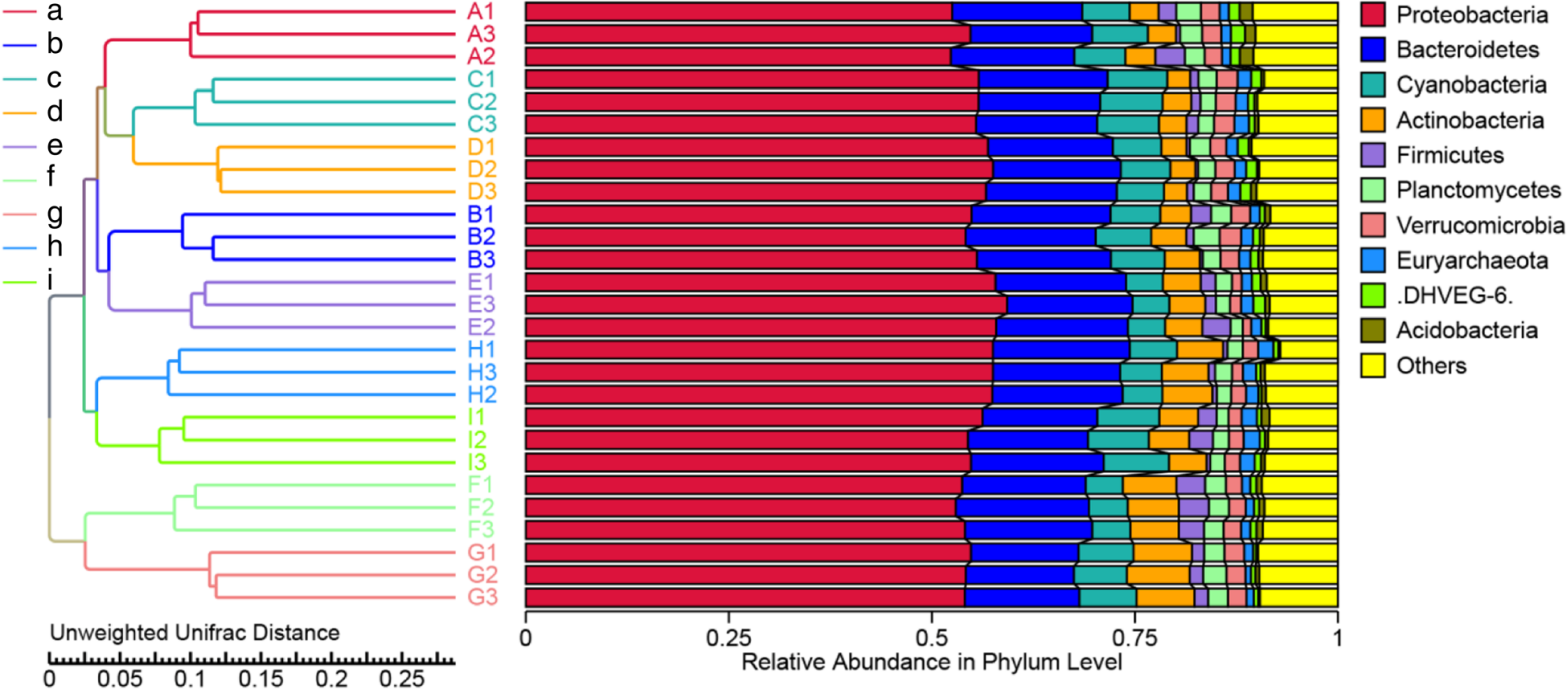
The UPGMA dendrogram of relative abundance at phylum level from three samplings of each sampling site. Site **a**, mangrove forest at Black Sand Beach; **b**, mangrove forest at Kungkrabaen Bay; **c**, tourist site at Suanson Beach; **d**, tourist site at Pattaya Beach; **e**, aquaculture site at Angsila old market; **f**, aquaculture site at Donhoylhod; **g**, aquaculture site at Bangtaboon Bay; **h**, mangrove forest at Pranburi forest park; **i**, tourist site at Wanakorn Beach

**Fig. 3:**
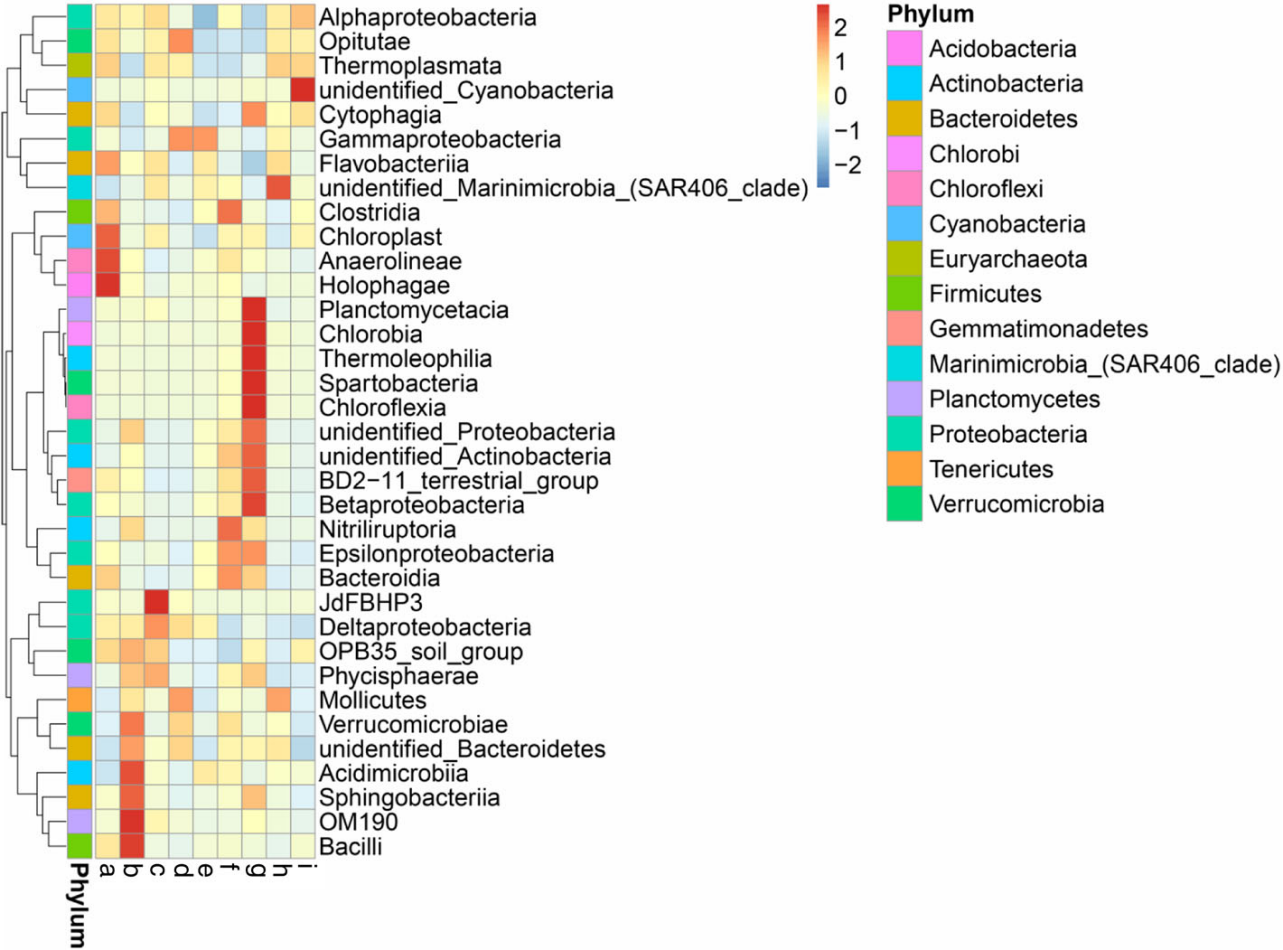
Heat map analysis of class distribution in each sampling site. Site **a**, mangrove forest at Black Sand Beach; **b**, mangrove forest at Kungkrabaen Bay; **c**, tourist site at Suanson Beach; **d**, tourist site at Pattaya Beach; **e**, aquaculture site at Angsila old market; **f**, aquaculture site at Donhoylhod; **g**, aquaculture site at Bangtaboon Bay; **h**, mangrove forest at Pranburi forest park; **i**, tourist site at Wanakorn Beach

The top ten most abundant phyla among nine sampling sites were in different patterns, as depicted in Fig. 2. The *Proteobacteria* were most abundant in all sampling sites, ranging between 67.31 and 78.80%, followed by the *Bacteroidetes* (10.38–17.68%), *Cyanobacteria* (2.64–15.16%), *Actinobacteria* (1.41–11.68%), *Verrucomicrobia* (0.30–1.89%), *Euryarchaeota* (0.24–1.47%), *Planctomycetes* (0.19–1.04%), *Marinimicrobia* (0.05–0.92%), *Firmicutes* (0.02–0.41%), Deep Sea Hydrothermal Vent Group 6 (DHVEG-6; 0.01–0.33%), and others (1.09–1.25%). The distribution of bacterial classes in each sampling site is shown in Fig. 3. The colors in a heat map chart indicate the relative abundance of the community. The colors which vary from deep blue to dark brown represent low- to high-levels of the relative abundance. The most abundant classes in each site are represented as dark-brown squares in a heat map chart. In site A (mangrove forest at Black Sand Beach), the *Holophagae*, *Anaerolineae*, and *Chloroplast* were more abundant than others. The *Bacilli*, OM190, *Sphingobacteriia*, *Acidimicrobiia*, and *Verrucomicrobiae* were the predominant classes in site B (mangrove forest at Kungkrabaen Bay). The greatest abundance of the JdFBHP3 was found in site C (tourist site at Suanson Beach). The *Mollicutes*, γ*-Proteobacteria*, and *Opitutae* were the predominant classes in site D (tourist site at Pattaya Beach). Site E (aquaculture site at Angsila old market) had the most abundance of the γ*-Proteobacteria*. Site F (aquaculture site at Donhoylhod) harbored the high numbers of the *Bacteroidia*, *Epsilonproteobacteria*, *Nitriliruptoria*, and *Clostridia*. Site G (aquaculture site at Bangtaboon Bay) had several most-abundant classes such as the *Chloroflexia, Spartobacteria*, *Thermoleophilia, Chlorobia*, and *Planctomycetacia*. The unidentified *Marinimicrobia* and unidentified *Cyanobacteria* were the most abundant classes in sites H (mangrove forest at Pranburi forest park) and I (tourist site at Wanakorn Beach), respectively.

The top ten most abundant genera present in each sampling site are shown in an Additional file 3: Table S1). The highest numbers of the genus *Marinobacterium* was presented in four sites including E (aquaculture site at Angsila old market), H (mangrove forest at Pranburi forest park), G (aquaculture site at Bangtaboon Bay), and A (mangrove forest at Black Sand Beach), which were not significantly different from each other. Site D (tourist site at Pattaya Beach) had the greatest number of the *Neptuniibacter*, differing significantly from that of other sites. Site I (tourist site at Wanakorn Beach) had the highest abundances of the *Synechococcus* and *Candidatus* Pelagibacter which were significantly different from those of other sites. The numbers of the *Candidatus* Actinomarina, *Candidatus* Thiobios, *Vibrio,* and *Marinomonas* were significantly highest in sites B (mangrove forest at Kungkrabaen Bay), G (aquaculture site at Bangtaboon Bay), C (tourist site at Suanson Beach), and E (aquaculture site at Angsila old market), respectively.

**Fig. 4:**
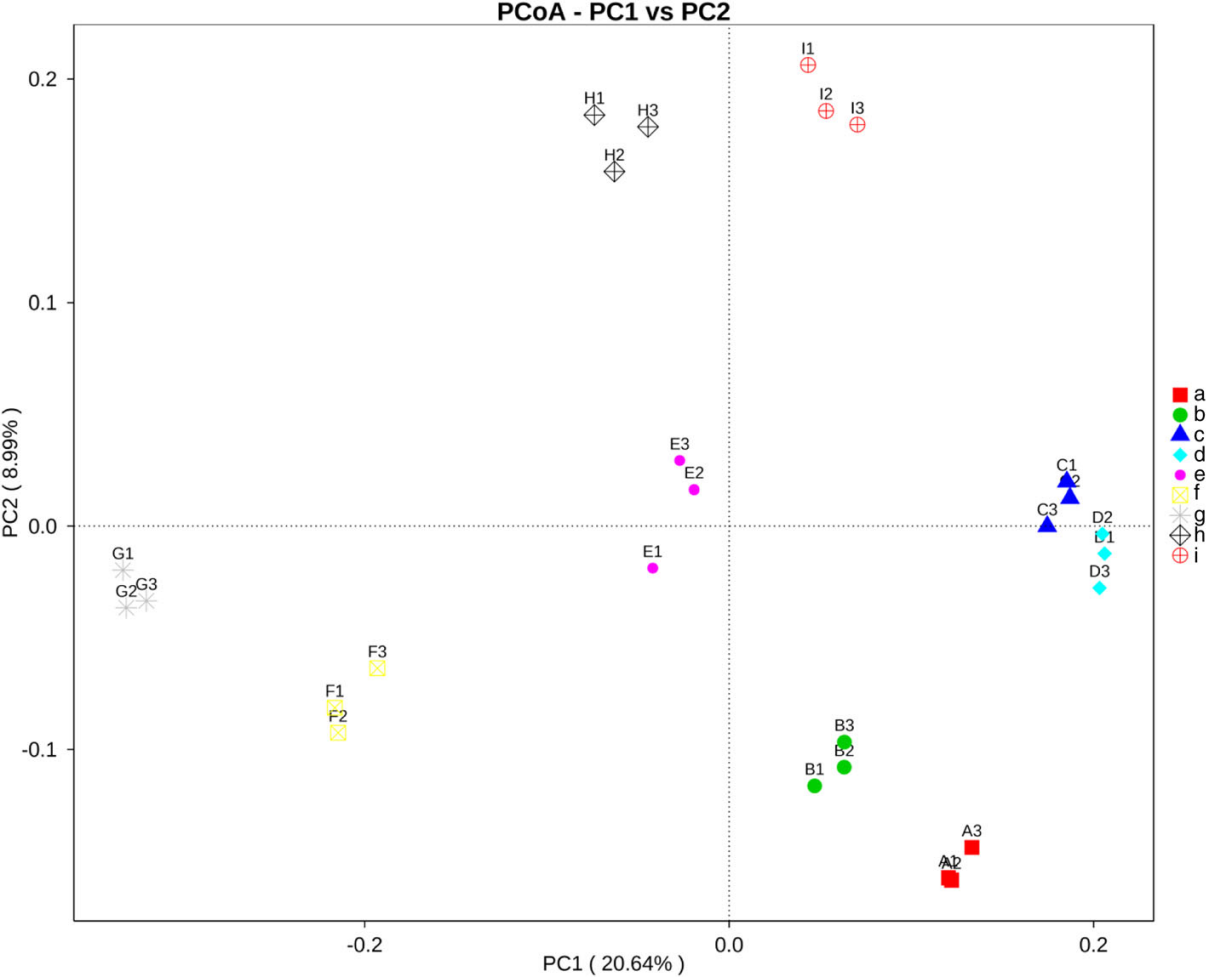
Principal Coordinate Analysis (PCoA) of species composition similarity by sampling sites. Site **a**, mangrove forest at Black Sand Beach; **b**, mangrove forest at Kungkrabaen Bay; **c**, tourist site at Suanson Beach; **d**, tourist site at Pattaya Beach; **e**, aquaculture site at Angsila old market; **f**, aquaculture site at Donhoylhod; **g**, aquaculture site at Bangtaboon Bay; **h**, mangrove forest at Pranburi forest park; **i**, tourist site at Wanakorn Beach

The ordination of samples by principal coordinate analysis (PCoA) shown in Fig. 4 revealed a significant clustering of samples by sampling site, and this separation was supported by analysis of molecular variance (AMOVA) (*P* < 0.001). Moreover, the variations in community composition among groups and within groups were evaluated by analysis of similarity (ANOSIM) and multi-response permutation procedure (MRPP). The results of both methods indicate that there were significant differences when comparing microbiota by sampling site (*P* < 0.05), and the variations of inter-group were larger than those of inner-group (*r* = 1). The unweighted-pair group method with arithmetic mean (UPGMA) dendrogram of the relative abundance at the phylum level depicted in Fig. 2 was divided into four clusters. The first cluster that contained sites A (mangrove forest at Black Sand Beach), C (tourist site at Suanson Beach), and D (tourist site at Pattaya Beach), was closer to the second cluster that contained sites B (mangrove forest at Kungkrabaen Bay) and E (aquaculture site at Angsila old market). The third cluster was composed of sites H (mangrove forest at Pranburi forest park) and I (tourist site at Wanakorn Beach). The last cluster containing sites F (aquaculture site at Donhoylhod) and G (aquaculture site at Bangtaboon Bay) was more separated from the other clusters.

### Effect of environmental factors on the bacterial communities

Effect of seawater physicochemical parameters on the bacterial communities was analyzed. The results show that members of the *α*-*Proteobacteria* and *Flavobacteriia* were positively associated with % NaCl (Spearman’s *r* = 0.434, *P* = 0.24; *r* = 0.63, *P* = 0.06) and negatively associated with TSS (*r* = − 0.68, *P* = 0.04; *r* = − 0.76, *P* = 0.01). The γ-*Proteobacteria* were also positively associated with % NaCl (*r* = 0.39, *P* = 0.30) and negatively associated with total P (*r* = − 0.38, *P* = 0.30). The members of β-*Proteobacteria*, unidentified *Proteobacteria*, and *Actinobacteria* were positively associated with total N (*r* = 0.88, *P* = 0.00; *r* = 0.88, *P* = 0.00; *r* = 0.94, *P* = 0.00) and negatively associated with % NaCl (*r* = − 0.73, *P* = 0.02; *r* = − 0.63, *P* = 0.06; *r* = − 0.73, *P* = 0.02). The *Cyanobacteria* were positively associated with total P (*r* = 0.49, *P* = 0.17) and negatively associated with BOD_5_ (*r* = − 0.46, *P* = 0.20). The *Acidimicrobiia* were positively associated with total N (*r* = 0.25, *P* = 0.50) and negatively associated with seawater temperature (*r* = − 0.52, *P* = 0.15). The δ*-Proteobacteria* were positively associated with BOD_5_ (*r* = 0.51, *P* = 0.15) and negatively associated with total N (*r* = − 0.59, *P* = 0.09). Moreover, in this study we also found that the numbers of the *Marinobacterium*, *Neptuniibacter*, *Synechococcus*, *Candidatus*Thiobios, hgcI clade (*Actinobacteria*), and *Candidatus* Pelagibacter were significantly different when grouped by land use (*P* = 0.00, 0.02, 0.02, 0.00, 0.03, and 0.00). The numbers of *Synechococcus* and *Candidatus* Pelagibacter were significantly higher in tourist sites than those in other types of land use, whereas *Candidatus* Thiobios was only one genus whose number was significantly higher in aquaculture sites than that in other types of land use (Additional file 4: Table S2).

### Discussion

Marine environment is the one of the most extensive habitats for microorganisms, covering more than two-thirds of the surface of the earth [20]. Marine bacteria play important roles in energy and matter fluxes in the sea [7]. Normal cell counts of more than 10^5^ cells/ml in surface seawater support the prediction that the oceans harbor 3.6 × 10^29^ microbial cells with a total cellular carbon content of approximately 3 × 10^17^ g [21], thereby an understanding of the marine bacterial distribution and diversity is essential. Although some studies investigated the microbial distribution and diversity with regard to the environmental and geographical conditions [6, 22, 23], the marine bacterial distribution and diversity regarding types of land use in the Gulf of Thailand have never been reported. In this study, we first investigated the communities and diversity of bacteria associated with seawater collected from three different types of land use including mangrove forests, tourist sites, and aquaculture sites, over a distance of approximately 769.97 km along the shores of the Upper Gulf of Thailand. The result shows that run-off water from each type of land use significantly affected the community richness and diversity of marine bacteria. Aquaculture sites contained the highest levels of community richness and diversity, followed by mangrove forests and tourist sites. The maximum richness and diversity in aquaculture sites possibly resulted from aquaculture activities such as feeding that increases the numbers of aquatic animals who are effective feeders promoting high levels of aquatic bacteria which are released from their feces and body fluids [24] and addition of readily accessible C source that significantly increases the bacterial biomass [25]. Mangrove forests are complex and dynamic ecosystems that are highly variable in several physicochemical conditions including salinity, flooding, light, temperature, and nutrient, which promote the bacterial diversity. It was reported that mangrove species were the main factors influencing their rhizosphere bacterial communities [26]. The run-off water in tourist sites may come from various sources such as swimmers, trash disposal from tourists, domestic wastewater, and illegal discharge from recreation boats [24]. Hamilton et al. [27] reported that pollutants from anthropogenic-influenced sources conveyed diverse bacteria into beaches and seawater.

Moreover, when determining the effect of environmental factors on the bacterial communities, we found that species richness estimators and OTUs were positively correlated with turbidity. Aquaculture sites had the highest average values of turbidity, TSS, and total N, which were significantly different from those of other types of land use. It can be concluded that a higher seawater turbidity level contributed to higher levels of species richness and OTUs. The positive and negative correlations between bacterial communities in class level and environmental factors were analyzed. The results show that the *α*-*Proteobacteria*, γ-*Proteobacteria*, and *Flavobacteriia* were positively associated with % NaCl. The *Cyanobacteria* were positively associated with total P. The members of β-*Proteobacteria*, unidentified *Proteobacteria*, *Acidimicrobiia*, and *Actinobacteria* were positively associated with total N. These results indicate that salinity, total N, and total P were the ones of the main factors shaping the bacterial communities of near-shore seawater in the Upper Gulf of Thailand, whereas pH and seawater temperature were not likely to affect the bacterial communities.

Our findings agree with that of Suh et al. [7] who studied seasonal dynamic of marine microbial community in the South Sea of Korea and found that salinity, N, and P contents contributed substantially to the spatial distribution of bacterial community composition. Salinity showed a marked correlation with the spatial distribution of the *Flavobacteriia*, while the *α*-*Proteobacteria* were greatly affected by N and dissolved oxygen. Likewise, the γ-*Proteobacteria* in seawater of Mallorca Island in Spain were positively correlated with salinity [12]. Inorganic nutrients were reported to importantly affect the bacterial community structures of seawater from the Mediterranean Sea, France, under euthrophication conditions [28] and natural seawater across Japan [6].

To more clearly study the similarity among bacterial communities in different sampling sites, UPGMA analysis was applied to display the integration and the relative abundance of each phylum in each site. The result shows that the relative abundance of bacterial phyla in seawater of sites F (aquaculture site at Donhoylhod) and G (aquaculture site at Bangtaboon Bay) was different from that of other sites. This may be affected by the physicochemical factors that shaped the bacterial communities of those two sites which ranked first and second in turbidity, TSS, and total N. Moreover, the proportions of the *Proteobacteria*, *Actinobacteria*, *Verrucomicrobia*, *Euryarchaeota*, and Deep Sea DHVEG-6 in both sites were more similar to each other than to other sites.

In this study, a total of 4953 OTUs were observed from all samples. Indeed, the amount of the bacterial OTUs was not necessarily correlated with location. The amounts of the bacterial OTUs in samples greatly varied depending on several physicochemical and environmental factors [29, 30]. Other studies revealed that the seawater collected from Gosung Bay (the South Sea of Korea) and Mallorca Island in Spain had only 900 OTUs [7] and 965 OTUs [12], respectively. The considerably more OTUs were observed in marine sediments. Totals of 6039 OTUs, 6059 OTUs, and 5700 to 7600 OTUs were obtained from marine sediments around the Kaichu-Doro Causeway in Okinawa, Japan [31], marine sediments in Yam O Wan Bay, Hong Kong [32], and marine sediments from Jeju Island, South Korea [20], respectively. Moreover, this study found that the proportions of the *Proteobacteria* were highest in all sampling sites, followed by the *Bacteroidetes* and *Actinobacteria*. This result corresponds with that of Suh et al. [7] who reported that the *Proteobacteria* was the dominant phylum in seawater from Gosung Bay, South Korea, followed by the *Bacteroidetes* and *Actinobacteria*. Similarly, most of the bacterial sequence reads in marine sediments from Jeju Island, South Korea, were also associated with the *Proteobacteria* and *Bacteroidetes*, followed by the *Actinobacteria*, *Acidobacteria*, and *Firmicutes* [20].

## Conclusions

This is the first report of the bacterial communities and diversity associated with seawater along the Upper Gulf of Thailand that was categorized into three types of land use including mangrove forests, tourist sites, and aquaculture sites. The run-off water from each type of land use significantly affected the community richness and diversity. The highest community richness and diversity were obtained from aquaculture sites, followed by mangrove forests and tourist sites. Turbidity was the most influential parameter affecting the variation in bacterial community composition. Salinity, total N, and P were the ones of the important factors that shaped the bacterial communities in near-shore seawater from the Upper Gulf of Thailand, whereas pH and seawater temperature less affected the bacterial communities. In addition, the variations of bacterial communities from site-to-site were greater than within-site. The *Proteobacteria*, *Bacteroidetes*, *Actinobacteria*, *Cyanobacteria*, *Verrucomicrobia*, *Euryarchaeota*, *Planctomycetes*, *Firmicutes*, Deep Sea DHVEG-6, and *Marinimicrobia* were the most and common phyla distributed across the Upper Gulf of Thailand.

## Methods

### Sample collection and determination of seawater parameters

Seawater was sampled on 10th November and 1st December 2018 at nine sites in seven provinces along the shores of the Upper Gulf of Thailand, over a distance of approximately 769.97 km (Table 1 and Fig. 1). Sampling sites were selected based on types of land use that were presumably influenced by different run-off conditions. Three sites (A, B, and H) were mangrove forests in Black Sand Beach, Kungkrabaen Bay, and Pranburi forest park, respectively. Three tourist sites (C, D, and I) were Suanson Beach, Pattaya Beach, and Wanakorn Beach, respectively. Three aquaculture sites (E, F, and G) were Angsila old market, Donhoylhod, and Bangtaboon Bay, respectively. All sampling sites were in public areas thus no specific permission was required for seawater collection. At each sampling site, near-surface seawater (12 L), approximately 2 m from the shoreline, was collected in triplicate and stored on ice during within-a-day transit.

Air and seawater temperatures were measured at each sampling site at the time of seawater collection. Seawater samples were analyzed for physicochemical parameters. pH, salinity, and turbidity (in NTU) were measured within 48 h by using a pH meter (Metrohm 827 pH lab), a hand refractometer (Atago N-1E), and a turbidimeter (HACH 2100P), respectively. BOD_5_, total N, total P, and TSS contents were analyzed according to American Public Health Association [33] by using the azide modification method, macro-Kjeldahl method, sulfuric acid-nitric acid digestion method, and drying at 103–105 °C, respectively.

### Illumina NGS

Seawater samples were prefiltered through sterile Whatman no. 2 filter papers to remove suspended particles and the filtrate was subsequently filtered through 0.2 μm sterile cellulose nitrate membrane filters (Sartorius, Stedim Biotech., Gottingen, Germany) [4]. DNA was extracted from seawater samples using E.Z.N.A^®^Water DNA kit (Omega Bio-tek, Inc., Norcross, GA, USA), according to the manufacturer’s instruction. The V4 variable region of the 16S rRNA gene was amplified by using the 515F and 806R specific primer set with the barcodes [34, 35]. PCR reactions were carried out with Phusion^®^ High-Fidelity PCR Master Mix (NEB, Ipswitch, MA, USA). The PCR products were purified using a Qiagen gel extraction kit (Qiagen, Inc., Valencia, CA, USA). The libraries were generated with TruSeq^®^ DNA PCR-Free sample preparation kit (Illumina, Inc., San Diego, CA, USA), and analyzed by HiSeq2500 PE250 sequencing system (Illumina, Inc., San Diego, CA, USA), according to the manufacturer’s instructions. Negative controls (sterile water) were carried out through amplification and sequencing. Data was returned as fastq files and deposited in the Sequence Read Archive of the National Center for Biotechnology Information under BioProject accession number PRJNA530863 (SRA: SRP190963).

### Data processing and bioinformatic analyses

Paired-end reads were merged by using the FLASH program (V1.2.7) [36]. Quality filtering on the raw tags was performed to obtain the high-quality clean tags according to the QIIME software (V1.7.0) [37, 38]. The tags were compared with the reference database using the UCHIME algorithm to detect chimera sequences. Chimera sequences were removed to obtain the effective tags [39, 40]. For OTU clustering and species annotation, sequence analysis was performed with all effective tags by using the Uparse software (V7.0.1001). Sequences with ≥97% similarity were assigned to the same OTUs. The Mothur software (V1.36.1) [41] was used to align each representative sequence against the SSU rRNA database of SILVA [42] for species annotation at each taxonomic level [43]. The phylogenetic relationship of all OTUs derived from representative sequences was analyzed by using the MUSCLE program (V3.8.31) [44].

### Statistical analyses

Alpha diversity, including community richness (Chao1 and ACE estimators), community diversity (Shannon and Simpson indices), and index of sequencing depth (the Good’ coverage) as well as rarefaction data were calculated by using the QIIME software (V1.7.0) and displayed by the R software (V2.15.3). PCoA was performed to obtain principal coordinates and visualize complex, multidimensional data, which was then displayed by the WGCNA, stat, and ggplot2 packages in the R software (V2.15.3). The UPGMA clustering was performed as a type of hierarchical clustering method to interpret the distance matrix using average linkage and conducted by using the QIIME software (V1.7.0). The nonparametic method, ANOSIM, was conducted to determine whether the bacterial community structures significantly differ among groups and within groups. MRPP was calculated with the R software (V2.15.3). AMOVA was performed using the Mothur software (V1.36.1). Seawater parameters, alpha diversity indices, and physicochemical parameters of each type of land use were subjected to an analysis of variance (ANOVA) using Tukey’s test. Spearman rank correlation was used to analyze the effect of seawater physicochemical parameters on the bacterial communities. ANOVA and Spearman rank correlations were performed with the SPSS statistical software (V19.0) (IBM Corp., Chicago, IL, USA). All statistical analyses were evaluated at α = 0.05.

## Supplementary information

**Additional file 1: Figure S1.** Rarefaction curves of observed species number from three samplings of each sampling site. Site A, mangrove forest at Black Sand Beach; B, mangrove forest at Kungkrabaen Bay; C, tourist site at Suanson Beach; D, tourist site at Pattaya Beach; E, aquaculture site at Angsila old market; F, aquaculture site at Donhoylhod; G, aquaculture site at Bangtaboon Bay; H, mangrove forest at Pranburi forest park; I, tourist site at Wanakorn Beach

**Additional file 2: Figure S2.** OTUs flower analysis of sampling sites.

**Additional file 3: Table S1.** Top ten most abundant genera present in each site. All sites were sampled in triplicate (*n* = 3). *Values are the means from three samplings ± standard deviations. **Values with the same letters within a column are not significantly different according to Tukey’s test.

**Additional file 4: Table S2.** Top ten most abundant genera present in each type of land use. *Values are the means of three samplings from each location ± standard deviations. **Values with the same letters within a column are not significantly different according to Tukey’s test. ***p-values < 0.05 are considered significant.

## Data Availability

All data generated or analyzed during this study has been included in this published article. Sequence data has been deposited in the Sequence Read Archive of the National Center for Biotechnology Information under BioProject accession number PRJNA530863 (SRA: SRP190963).

## Abbreviations

ACE: Abundance-based coverage estimator
AMOVA: Analysis of molecular variance;
ANOSIM: Analysis of similarity
ANOVA: Analysis of variance
BOD_5_: Five-day biochemical oxygen demand
MRPP: Multi-response permutation procedure
NGS: Next-generation sequencing
NTU: Nephelometric turbidity unit
OTU: Operational taxonomic unit
PCoA: Principal coordinate analysis
TSS: Total suspended solid
UPGMA: Unweighted-pair group method with arithmetic mean
