# Supplementary figures and images for: Marine bacterial communities in the upper gulf of Thailand assessed by Illumina next-generation sequencing platform
Source: BMC Microbiol. 2020 Jan 23;20:19. doi: 10.1186/s12866-020-1701-6 (PMC6979385; doi:10.1186/s12866-020-1701-6)

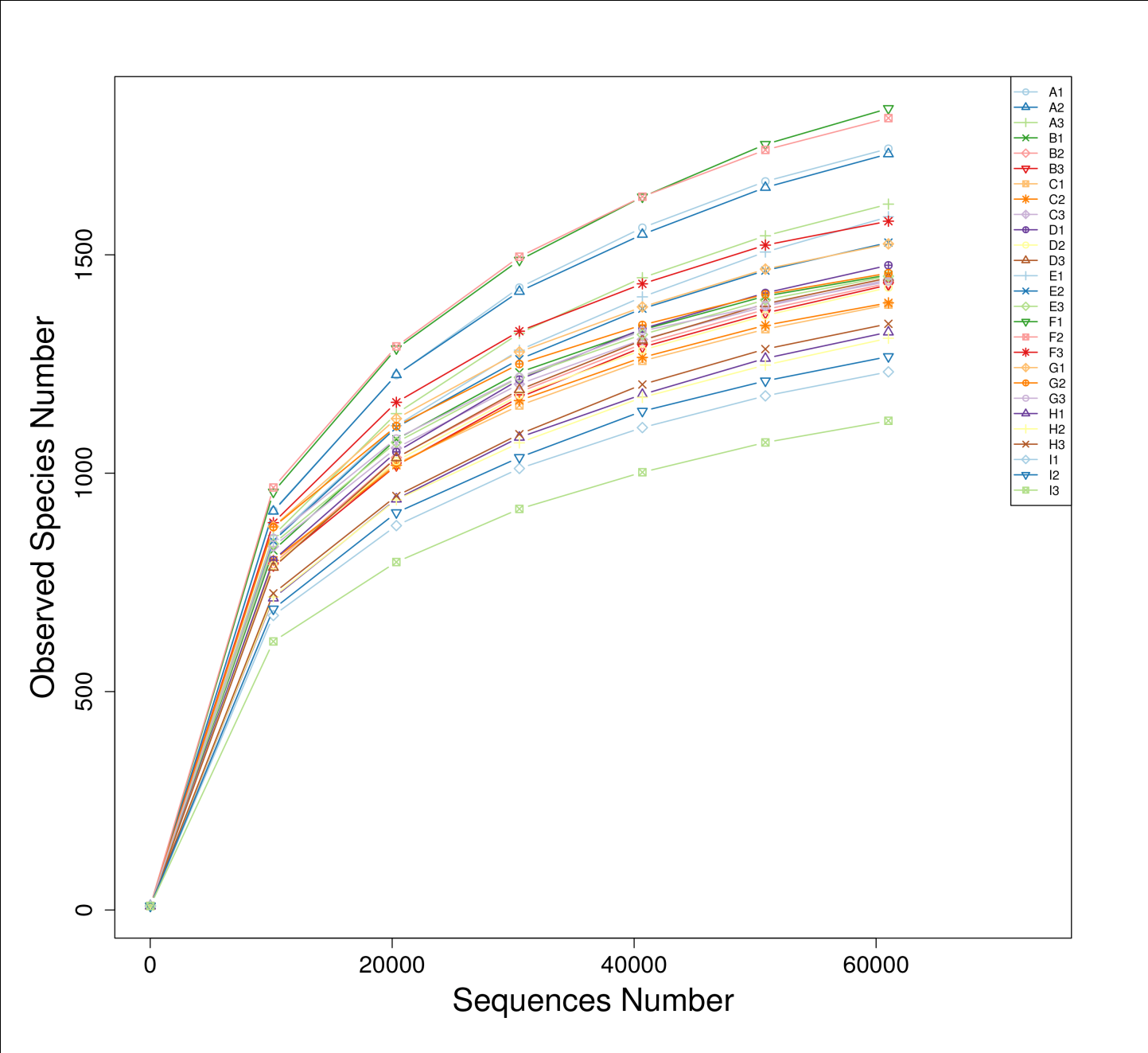

Supplement: Supplementary file 1 — Additional file 1: Figure S1. Rarefaction curves of observed species number from three samplings of each sampling site. Site A, mangrove forest at Black Sand Beach; B, mangrove forest at Kungkrabaen Bay; C, tourist site at Suanson Beach; D, tourist site at Pattaya Beach; E, aquaculture site at Angsila old market; F, aquaculture site at Donhoylhod; G, aquaculture site at Bangtaboon Bay; H, mangrove forest at Pranburi forest park; I, tourist site at Wanakorn Beach [file 12866_2020_1701_MOESM1_ESM.docx]

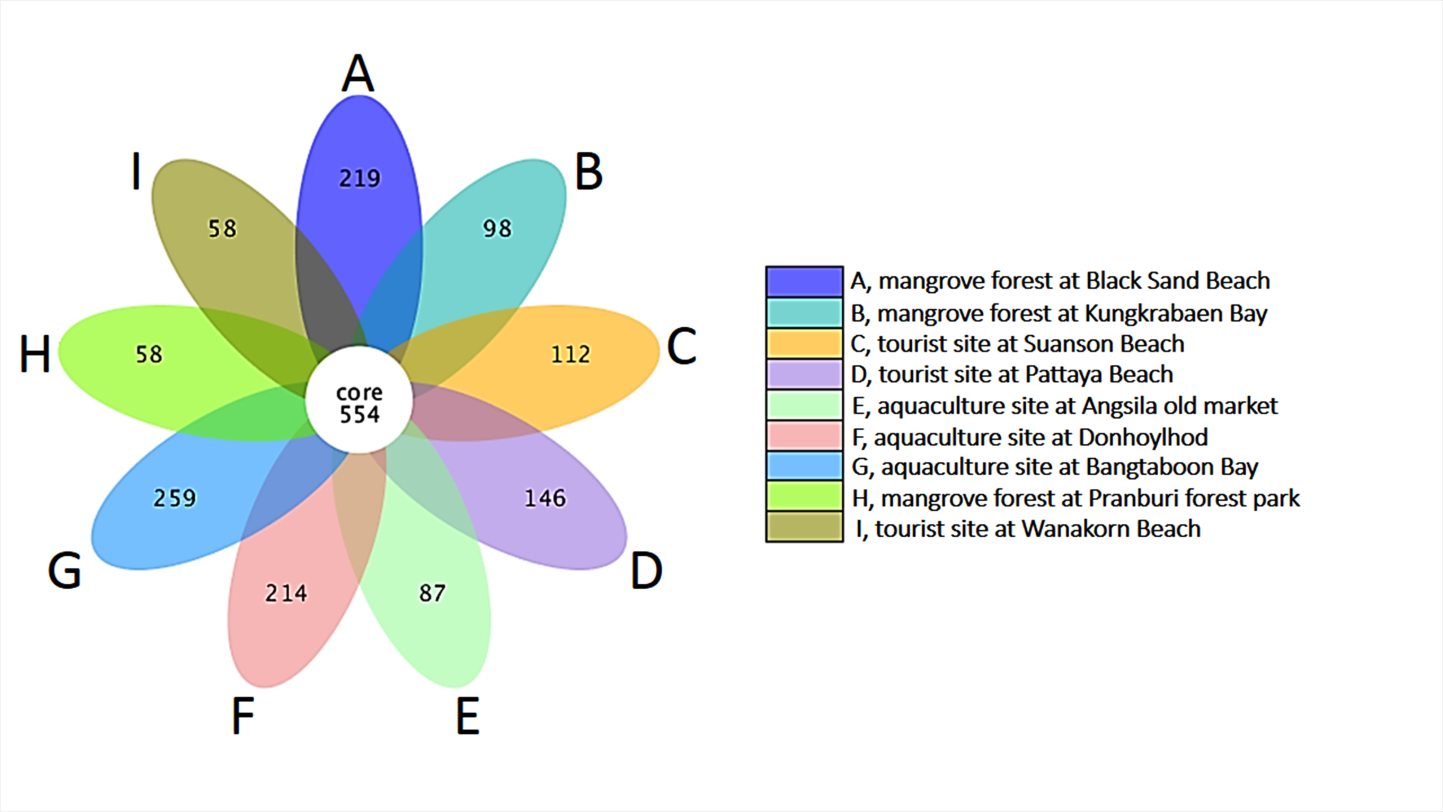

Supplement: Supplementary file 2 — Additional file 2: Figure S2. OTUs flower analysis of sampling sites. [file 12866_2020_1701_MOESM2_ESM.docx]
