# Supplementary material for: Marine bacterial communities in the upper gulf of Thailand assessed by Illumina next-generation sequencing platform
Source: BMC Microbiol. 2020 Jan 23;20:19. doi: 10.1186/s12866-020-1701-6 (PMC6979385; doi:10.1186/s12866-020-1701-6)
Supplement: Supplementary file 3 — Additional file 3: Table S1. Top ten most abundant genera present in each site. All sites were sampled in triplicate (n = 3). *Values are the means from three samplings ± standard deviations. **Values with the same letters within a column are not significantly different according to Tukey’s test. [file 12866_2020_1701_MOESM3_ESM.docx]

Additional file 3: **Table S1.** Top ten most abundant genera present in each site

| Site | Genus* | | | | | | | | | |
| --- | --- | --- | --- | --- | --- | --- | --- | --- | --- | --- |
|  | ***Marinobacterium*** | ***Neptuniibacter*** | ***Synechococcus*** | ***Candidatus***  **Actinomarina** | **NS5 marine group** | ***Candidatus***  **Thiobios** | ***Vibrio*** | ***Marinomonas*** | **hgcI_clade** | ***Candidatus***  **Pelagibacter** |
| A | 7.52± 2.45bc** | 0.08 ± 0.00c | 0.96 ± 0.20a | 1.66 ± 0.14a | 5.42 ± 0.77bcd | 0.30 ± 0.01a | 1.75 ± 0.21c | 0.12 ± 0.02a | 0.00a | 0.95±0.03a |
| B | 2.79± 0.08ab | 0.18 ± 0.01e | 0.85 ± 0.09a | 9.66 ± 0.15e | 6.68 ± 0.80d | 3.93 ± 0.29d | 0.41 ± 0.08a | 0.03 ± 0.00a | 0.02 ± 0.00c | 2.57±0.27d |
| C | 0.96 ± 0.20a | 0.36 ± 0.08f | 1.73 ± 0.46a | 3.76 ± 0.37bc | 6.47 ± 0.48cd | 0.02 ± 0.00a | 5.26 ± 0.51e | 1.66 ± 0.21b | 0.00a | 2.48±0.28cd |
| D | 1.05 ± 0.43a | 16.60 ± 0.53g | 0.74 ± 0.17a | 2.37 ± 0.39ab | 2.48 ± 0.15a | 0.04 ± 0.02a | 0.61 ± 0.12ab | 0.13 ± 0.01a | 0.00a | 2.00±0.19bc |
| E | 13.85 ± 0.57c | 0.09 ± 0.00d | 0.75 ± 0.07a | 5.35 ± 0.41d | 6.58 ± 0.67d | 1.36 ± 0.06b | 2.95 ± 0.21d | 4.51 ± 0.61c | 0.01 ± 0.00b | 2.16±0.13bcd |
| F | 4.78 ± 1.49ab | 0.05 ± 0.00b | 1.06 ± 0.11a | 4.71 ± 1.11cd | 4.50 ± 1.24abc | 2.80 ± 0.30c | 0.48 ± 0.04a | 0.14 ± 0.00a | 0.13 ± 0.04e | 0.83±0.04a |
| G | 9.00 ± 1.22bc | 0.00a | 1.16 ± 0.14a | 2.91 ± 0.19ab | 3.09 ± 0.65a | 6.15 ± 0.33e | 0.11 ± 0.00a | 0.04 ± 0.01a | 3.44 ± 1.27f | 1.71±0.10b |
| H | 13.38 ± 5.88c | 0.00a | 1.15 ± 0.25a | 3.70 ± 0.40bc | 3.75 ± 0.69ab | 0.15 ± 0.01a | 0.66 ± 0.16ab | 0.28 ± 0.03a | 0.07 ± 0.01d | 0.81±0.18a |
| I | 0.34 ± 0.10a | 0.00a | 10.10 ± 1.32b | 3.51 ± 0.61bc | 3.63 ± 0.50ab | 0.03 ± 0.01a | 1.12 ± 0.06b | 0.04 ± 0.00a | 0.00a | 3.87±0.31e |

All sites were sampled in triplicate (*n* = 3).

*Values are the means from three samplings ± standard deviations.

**Values with the same letters within a column are not significantly different according to Tukey’s test.
