# Supplementary material for: Marine bacterial communities in the upper gulf of Thailand assessed by Illumina next-generation sequencing platform
Source: BMC Microbiol. 2020 Jan 23;20:19. doi: 10.1186/s12866-020-1701-6 (PMC6979385; doi:10.1186/s12866-020-1701-6)
Supplement: Supplementary file 4 — Additional file 4: Table S2. Top ten most abundant genera present in each type of land use. *Values are the means of three samplings from each location ± standard deviations. **Values with the same letters within a column are not significantly different according to Tukey’s test. ***p-values < 0.05 are considered significant. [file 12866_2020_1701_MOESM4_ESM.docx]

Additional file 4: **Table S2.** The top ten most abundant genera present in each type of land use

| Type of land use | Genus* | | | | | | | | | |
| --- | --- | --- | --- | --- | --- | --- | --- | --- | --- | --- |
|  | ***Marinobacterium*** | ***Neptuniibacter*** | ***Synechococcus*** | ***Candidatus***  **Actinomarina** | **NS5 marine group** | ***Candidatus***  **Thiobios** | ***Vibrio*** | ***Marinomonas*** | **hgcI_clade** | ***Candidatus***  **Pelagibacter** |
| Mangrove forests | 7.90 ± 5.59b** | 0.09 ± 0.07ab | 0.98 ± 0.21a | 5.01 ± 3.60a | 5.28 ± 1.43a | 1.46 ± 1.86a | 0.94±0.62a | 0.14 ± 0.11a | 0.03 ± 0.33b | 1.44 ± 0.86a |
| Tourist sites | 0.78 ± 0.41a | 5.65 ± 8.21b | 4.19 ± 4.50b | 3.21 ± 0.76a | 4.19 ± 1.81a | 0.03 ± 0.01a | 2.33 ± 2.22a | 0.61 ± 0.79a | 0.00a | 2.78 ± 0.87b |
| Aquaculture sites | 9.21 ± 4.05b | 0.49 ± 0.41a | 0.99 ± 0.21a | 4.33 ± 1.25a | 4.73 ± 1.70a | 3.44 ± 2.14b | 1.18 ± 1.34a | 1.56 ± 2.23a | 1.20 ± 1.80b | 1.93 ±0.97a |
| p-value***  (between groups) | 0.00 | 0.02 | 0.02 | 0.25 | 0.39 | 0.01 | 0.14 | 0.10 | 0.03 | 0.00 |

*Values are the means of three samplings from each location ± standard deviations.

**Values with the same letters within a column are not significantly different according to Tukey’s test.

***p-values < 0.05 are considered significant.
